# Removal of As(V) and Cr(VI) using quinoxaline chitosan schiff base: synthesis, characterization and adsorption mechanism

**DOI:** 10.1186/s13065-024-01328-7

**Published:** 2024-11-11

**Authors:** Huda E. Abdelwahab, Mohammed Elhag, Mohamed M. El Sadek

**Affiliations:** 1 Chemistry Department, Faculty of Science, Alexandria University, Alexandria, 21231 Egypt; 2 Institute of Graduate Studies and Research (IGSR), Alexandria University, Alexandria, 21526 Egypt; 3 Chemistry Department, Faculty of Science, Damanhour University, Damanhour, 22511 Egypt

**Keywords:** Chitosan, Schiff base, Water treatment, As(V) removal, Cr(VI) removal, Adsorption

## Abstract

Elevated Arsenic and Chromium levels in surface and ground waters are a significant health concern in several parts of the world. Chitosan quinoxaline Schiff base (CsQ) and cross-linked chitosan quinoxaline Schiff base (CsQG) were prepared to adsorb both Arsenate [As(V)] and Chromium [Cr(VI)] ions. The thermo-gravimetric analysis (TGA), X-ray diffraction analysis (XRD), and Fourier-transform infrared spectroscopy (FTIR) were used to investigate the prepared Schiff bases (CsQ) and (CsQG). The UV–VIS spectra showed a shift in the wavelength area of the modified polymer, indicating the reaction occurrence, besides the variation of the shape and intensity of the peaks. The XRD patterns showed the incensement of the amorphous characteristic. On the other hand, the thermal stability of the modified polymers is better according to TGA studies; also, the morphology of the modified chitosan was investigated before and after crosslinking (CsQ and CsQG) using a scanning electron microscope (SEM) where the surface was fall of wrinkles and pores, which then were decreased after cross-linking. Contact time, temperature, pH, and initial metal ion concentration were all studied as factors influencing metal ion uptake behavior. The Langmuir, Temkin, Dubinin–Radushkevich, and Freundlich isotherm models were used to describe the equilibrium data using metal concentrations of 10–1000 mg/L at pH = 7 and 1 g of adsorbent. The pseudo-first-order and pseudo-second-order kinetic parameters were evaluated. The experimental data revealed that the adsorption kinetics follow the mechanism of the pseudo-second-order equation with R^2^ values (0.9969, 0.9061) in case of using CsQ and R^2^ values (0.9989, 0.9999) in case of using CsQG, demonstrating chemical sorption is the rate-limiting step of the adsorption mechanism. Comparing the adsorption efficiency of the synthesized Schiff base and the cross-linked one, it was found that CsQ is a better adsorbent than CsQG in both cases of As(V) and Cr(VI) removal. This means that cross-linking doesn’t enhance the efficiency as expected, but on the contrary, in some cases, it decreases the removal. In addition, the newly modified chitosan polymers work better in As(V) removal than Cr(VI); the removal is 22.33% for Cr(VI) and 98.36% for As(V) using CsQ polymer, whereas using CsQG, the values are 6.20% and 91.75% respectively. On the other hand, the maximum adsorption capacity (Qm) for As(V) and Cr(VI) are 8.811 and 3.003 mg/g, respectively, using CsQ, while in the case of using CsQG, the Qm value reaches 31.95 mg/g for As(V), and 103.09 mg/g for Cr(VI).

## Introduction

Massive volumes of heavy metal ions have been routinely released into the environment in recent years. They originate from various industries' waste materials, including metallurgy, glass processing, electroplating, leather tanning, paints and pigments, textile production, and steel manufacturing, as well as natural sources like rocks [1, 2]. Heavy metals have several disadvantages and risks, including being extremely poisonous and dangerous to human health [3]. The most dangerous compounds to avoid are chromium [Cr(VI)] and arsenate [As(V)] [4]. The International Agency for Research on Cancer (IARC) has categorized all Cr(VI) compounds and arsenic as group 1 as carcinogens [5]. Chromium exists in the environment as two valence states: hexavalent chromium [Cr(VI)] and trivalent chromium [Cr(III)]. Cr(VI) is about 500 or 1000 times more toxic than Cr(III) and is more soluble in watery and soil environments [6]. Prolonged exposure to chromium and arsenic-contaminated water has been known to cause cancers of the kidney, liver, prostate, bladder, and lungs, as well as diabetes mellitus, oxidative stress, and hypertension [7–10]. Other diseases associated with extended exposure to arsenic-contaminated water include dermal lesions, hyperkeratosis, peripheral neuropathy, loss of appetite, and limb gangrene [11, 12]. Furthermore, aquatic organisms exposed to low amounts of chromium and arsenic have been linked to various cancers and mutagenesis [13].

According to the World Health Organization (WHO), the limits for chromium and arsenic in drinking water are 0.05 and 0.01 ppm, respectively. [14, 15] Consequently, lowering the concentrations of chromium and arsenic in the water below these limits is a challenge. Therefore, the extreme toxicity of chromium and arsenic has prompted researchers worldwide to investigate new technical methods for removing them from drinking water economically and efficiently. Reverse osmosis, foam-flotation, solvent extraction, coagulation/precipitation, filtration, sedimentation, solvent extraction, coagulation/precipitation, electrolysis, and membrane processes are conventional methods used to remove chromium, arsenic, and, in general, heavy metals [6, 16–20]. Most of these removal methods have several defects, such as inefficiency, time-consuming, and relatively expensive, and their efficacy varies largely based on the pH of the metal ion solution. As a result, the need for a proper removal process that is fast, cheap, and effective across a broad pH range is critical. The most prevalent straightforward method to accomplish such a removal process is adsorption. It has been used a lot to remove toxic metal ions and fulfill the required criteria [21]. Adsorption, compared to other purification techniques like chemical precipitation, membrane filtration, and ion exchange, has advantages like the wide accessibility of adsorbents, cost-effectiveness, high removal efficiency at very low concentrations, high selectivity, efficiency, environmental friendliness [20], and ease of operation [19].

How the adsorbent was chosen is very important; it depends on some important fetchers such as cost, source availability, safety, recoverability, and efficiency. For this purpose, using low-cost, non-toxic, and abundant adsorbents in nature has become widespread in recent years, such as natural polymers [22]. Chitosan, a non-toxic, biodegradable linear cationic biopolymer consisting of β-(1–4) D-glucopyranosamine repeating units, is recommended for biomedical and agricultural applications. Chitosan contains plentiful adsorption sites represented in amino (-NH_2_) and hydroxyl (-OH) groups, particularly the amino groups, which interact effectively with metal ions [23]. As a result, chitosan is one of the most effective biopolymers for eliminating heavy metals [24], specifically chromium and arsenic, from wastewater [25–27]. Furthermore, chitosan applicability is restricted because of its solubility in acidic solutions. Consequently, several chitosan modifications have been accomplished by introducing additional functional groups onto the chitosan backbone. Such incorporation of additional functional groups increases the adsorption sites number, alters the range of pH for metal sorption, and improves the sorption selectivity for the targeted metal [28]. Recent studies showed that due to the advantage of chitosan, it can be used as both a chelating agent and a flocculant. For instance, several chitosan-based composites were synthesized and investigated [27], several cross-linking agents were used, and incorporation of new functional groups into chitosan derivatives (such as histidine, heparin, succinic anhydride, and N, O carboxymethyl), ultimately aimed at improving their adsorption ability and metal ion sorption selectivity [29]. Chitosan’s imine functionalization is one of the recognized chemical modifications that result in the formation of the well-known Schiff base. Usually, chitosan Schiff bases are formed by the facile condensation of chitosan's amino groups with aldehydes or ketones [30]. These Schiff bases have enormous potential for usage as adsorbents, particularly in contaminated effluent treatment [31].

Chitosan cross-linking is accomplished by carefully selecting dialdehydes such as glutaraldehyde [32], glyoxal [33], poly (ethylene glycol) dialdehyde [34], crown ether containing dialdehydes [35], dialdehyde alginate [36], and dialdehyde cellulose [37]. Glutaraldehyde is currently the most widely used cross-linker, with chitosan over others due to its high-water solubility; aqueous media can directly undergo cross-linking reactions with various cross-linking degrees. Glutaraldehyde can also be used for cross-linking under mild conditions, such as alkaline, neutral, and especially acidic solutions, where the chitosan must first be dissolved in diluted acetic acid. In addition, no additives, such as initiators or catalysts, are needed for glutaraldehyde cross-linking [28].

Recently, a focus has been on enhancing chitosan's stability and adsorption potential in acidic conditions through cross-linking and chitosan-Schiff base synthesis. For example, Abou El-Reash et al. [38] synthesized cross-linked magnetic chitosan anthranilic acid glutaraldehyde Schiff’s base (CAGS) as a very effective adsorbent for removing both As(V) and Cr(VI). Anush et al. [39] achieved a newly pyrazolo-chitosan Schiff base cross-linked with epichlorohydrin, incorporated with Fe3O4 nanoparticles, and investigated its adsorptive removal of Cu(II) and Cr(VI). Using oxidized sodium alginate and boric acid as cross-linking agents, Cao et al. [40] proposed a promising Cr(VI) adsorbent of double cross-linked dual self-healing hydroxypropyl chitosan hydrogel. Maity et al. [41] developed a selective bio-adsorbent furfuraldehyde–chitosan cross-linked hydrogel for As(V) removal from groundwater.

As a result, the current study aims to synthesize new low-cost adsorbent starting from chitosan as a cheap and available procure. Modifying chitosan via Schiff base formation and cross-linking in order to improve both acid resistance and chitosan's ability to adsorb As(V) and Cr(VI) ions. According to the literature review, it is the first study on the removal of As(V) and Cr(VI) ions using a cross-linked Schiff base of chitosan, where the used aldehyde contains Quinoxaline moiety. Quinoxaline-2-carbaldehyde and glutaraldehyde were precursors to create the Schiff base and cross-linking, respectively. The variables influencing the removal behavior will be studied. Additionally, kinetic and thermodynamic processes will be explained.

## Materials and methods

### Materials

Chitosan with an average molecular weight of (100,000–300,000 Da) and an 88 percent deacetylation degree (Acros Organics, Morris Plains, NJ, USA). Glutaraldehyde was purchased from Merck in a 25% solution in water. Sodium arsenate dibasic heptahydrate (Na_2_HAsO_4_·7H_2_O, 98.5%), potassium dichromate (K_2_Cr_2_O_7_, 99.2%), acetic acid (100%) and ether (99.9%) were supplied from Sigma-Aldrich. Ethanol (97.5%) as a solvent was supplied from Al-Nasr Co., Helwan, Egypt. All of the chemical reagents used are of analytical grade and used without any modification. The working solution was prepared using double distilled water.

### Measurements

The Perkin Elmer—USA Spectrometer was used to record the Fourier transform infrared spectra (FTIR) using KBr discs at 25 °C in the range of ƛ (4000–400) cm^−1^. All compounds were scanned using Evolution 300 UV–VIS in the range ƛ (200–1100) cm^−1^.

X-ray diffraction analysis (XRD) of the parent compound and the target compounds were recorded within the range of 2θ (10°–90°) at 5° min^−1^ as a scanning rate using a Germany powder X-ray diffractometer, Bruker; Advanced D_8_ model, with source 2.2 kW Cu anode.

Thermo-gravimetric analysis (TGA) was performed using the Simultaneous TGA-DTA Thermal Analyzer System (SDT 2960). All measurements were performed under a dynamic nitrogen atmosphere in aluminum pans (samples weighing ≈ 6–10 mg) with a scanning rate of 10 °C/min, in the temperature range (25–500)  °C, and at 100 mL/min gas flow. The solid residues were cooled at room temperature.

The morphology of the modified chitosan derivatives before and after Cr(VI) and As(V) adsorption was performed using a JEOL-JSM 5300 Scanning electron microscope (JEOL, Tokyo, Japan).

Thermo Scientific's atomic adsorption spectrometer (S series) was used to detect the concentration of As(V) and Cr(VI) solutions. An Adwa (AD—1000) pH meter was used to measure the pH of the prepared solutions.

### Synthesis of quinoxaline-2-carbaldehyde

Quinoxaline-2-carbaldehyde was prepared according to the methods described in Alyaninezhad et al. [42]. Drop-wise addition of sodium meta per-iodate solution (60 mmol in 75 ml H_2_O) to the corresponding C-nucleoside analog suspension (20 mmol in 200 ml H_2_O) was performed, followed by stirring for 3h in the dark at 25 °C. The aldehyde that separated was filtered off, washed with water, dried, and recrystallized from methanol as a colorless needle m.p. 107 °C (Lit. [46], 107 °C).

### Synthesis of chitosan quinoxaline Schiff base (CsQ)

A solution of quinoxaline-2-carbaldehyde (5 mmol) in ethanol (30 mL) was added gradually to chitosan solution (5 mmol dissolved in 100 mL of 2% (v/v) acetic acid), then diluted with (30 ml) ethanol, followed by a 5 h reflux of the resulting mixture with stirring, and then left to cool [43]. To remove the excess aldehyde, several washes using diethyl ether were performed. The resulting hydrogel was dried for 24 h under vacuum at 60 °C.

### Synthesis of cross-linked chitosan quinoxaline Schiff base (CsQG)

First, the CsQ Schiff base was synthesized as in the previous step as a pale-yellow hydrogel. Then, 2 ml of glutaraldehyde solution was diluted with 20 ml ethanol and added gradually above the CsQ solution under stirring overnight. Finally, the synthesized CsQG was washed several times with ether and dried in a vacuum at 60 °C for 24 h.

### Preparation of the Stock Solution

Potassium dichromate (K_2_Cr_2_O_7_, 2.825 g) was dissolved in distilled water (1000 mL) for the preparation of the stock solution (1000 mg/L) of Cr(VI) ion. A stock solution (1000 mg/L) was prepared in a volumetric flask by dissolving (4.16 g) of Na_2_HAsO_4_.7H_2_O in distilled water (1000 mL). The solution pH was adjusted using hydrochloric acid and sodium hydroxide.

### Adsorption Experiments

Sorption experiments were conducted using 100 mL of the prepared solutions. They weighed CsQ and CsQG as adsorbent polymers in dark glass bottles and used an orbit shaker for agitation at 250 rpm. All experiments were performed at room temperature.

The atomic adsorption spectrophotometer (AAS) detected the solution concentrations. The metal ions removal percentage was calculated using the following Eq.  [46]:

1 $$Removal \% =\frac{\left({C}_{0}- \complement \right)}{{C}_{0}}\times 100$$

where ${C}_{0}$ is the initial metal ion concentration (mg/L) and $\complement$ is the final metal ion concentration (mg/L).

The initial pH effect on the adsorption capacity of the modified polymer was studied by varying pH (2 -10) using an initial metal concentration of 1000 mg/L and polymer dosage (1g/100 mL) for 60 min contact time. The sorbent dosage effect was studied from 1 to 250 mg for one hour as contact time.

Studying the initial concentration effect on the adsorption capacity was conducted using 100 mL of the synthetic metal solutions with initial concentrations (5, 10, 15, 25, 50, 75, 100, and 200 mg/L). The effect of contact time was studied in the 10–450 min range and at 1000 mg/L as initial metal concentrations. Adsorption isotherms were studied at 25 °C with different initial concentrations of chromium and arsenate.

## Results and discussion

### cross-linked chitosan Schiff bases synthesis

Several studies have been conducted to maximize the efficiency of cross-linking polymers for arsenic and chromium adsorption from wastewater. These studies have revealed that the type of polymer used, the pH of the water, and the initial metal concentration significantly affect the removal's efficiency. The introduction of glutaraldehyde into a polymer net can be beneficial in terms of enhancing the polymer's adsorption activity [27, 44]Glutaraldehyde has been studied for its potential to increase the adsorption activity of polymeric materials, and its introduction can indeed improve adsorption activity. [28]

Glutaraldehyde is a chemical compound that acts as a cross-linking agent for polymers. When added to a polymer net, it can form a three-dimensional structure that increases the number of interactions between the polymeric material and the adsorbent, thus increasing the overall adsorption activity of the polymeric material. Studies have shown that increasing the concentration of glutaraldehyde can lead to a greater degree of cross-linking, which can result in an even greater increase in the adsorption activity of the polymeric material. [32].

**Scheme 1.**
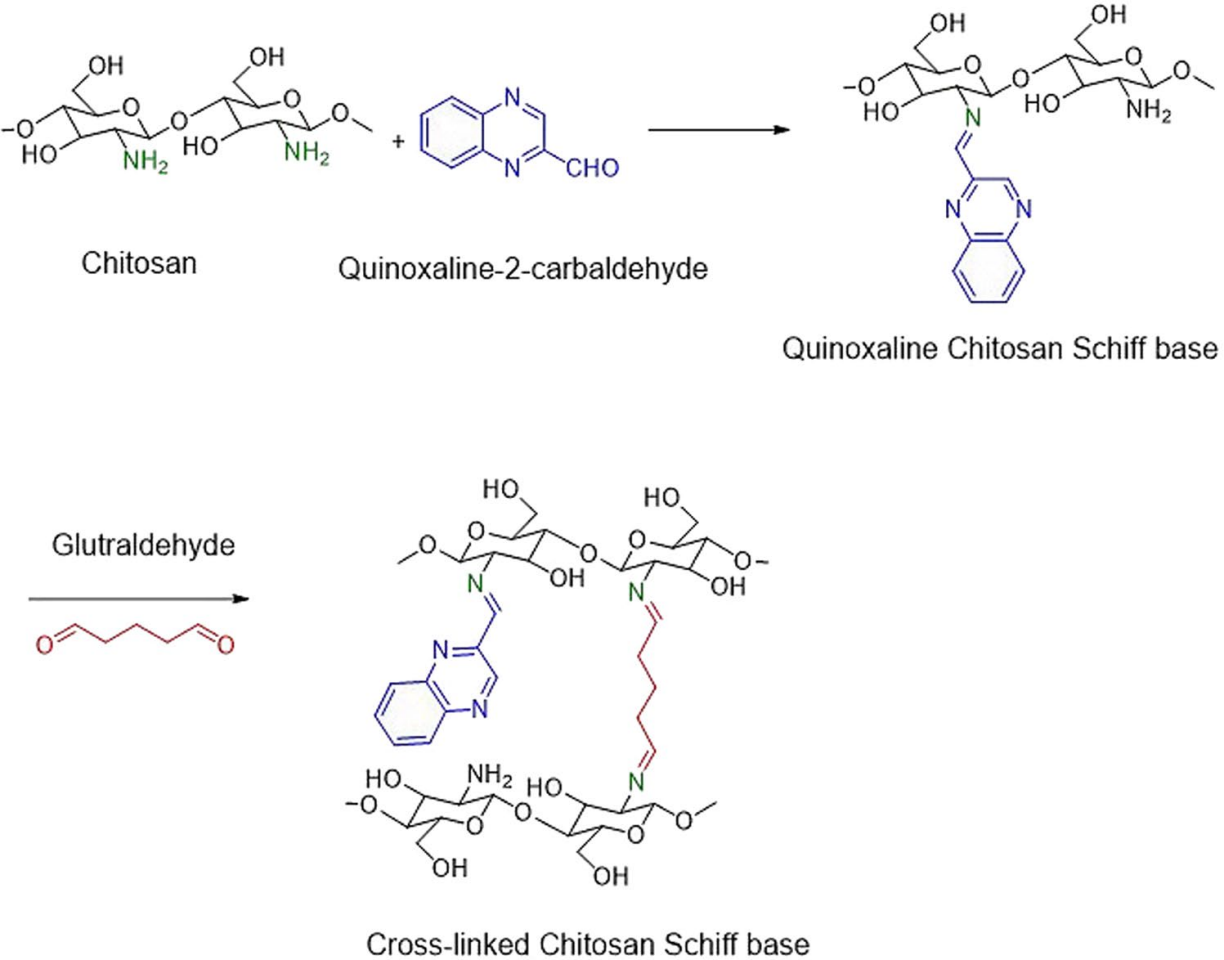
Synthesis of CsQ and CsQG

In the present work, the synthesis of cross-linked chitosan Schiff base started with Schiff's base formation between chitosan's amino group and quinoxaline-2-carbaldehyde’s active aldehydic group, as shown in Scheme 1. The produced chitosan quinoxaline Schiff base (CsQ) was subsequently cross-linked with glutaraldehyde cross-linker.

### Characterization of chitosan Schiff base

**Fig. 1:**
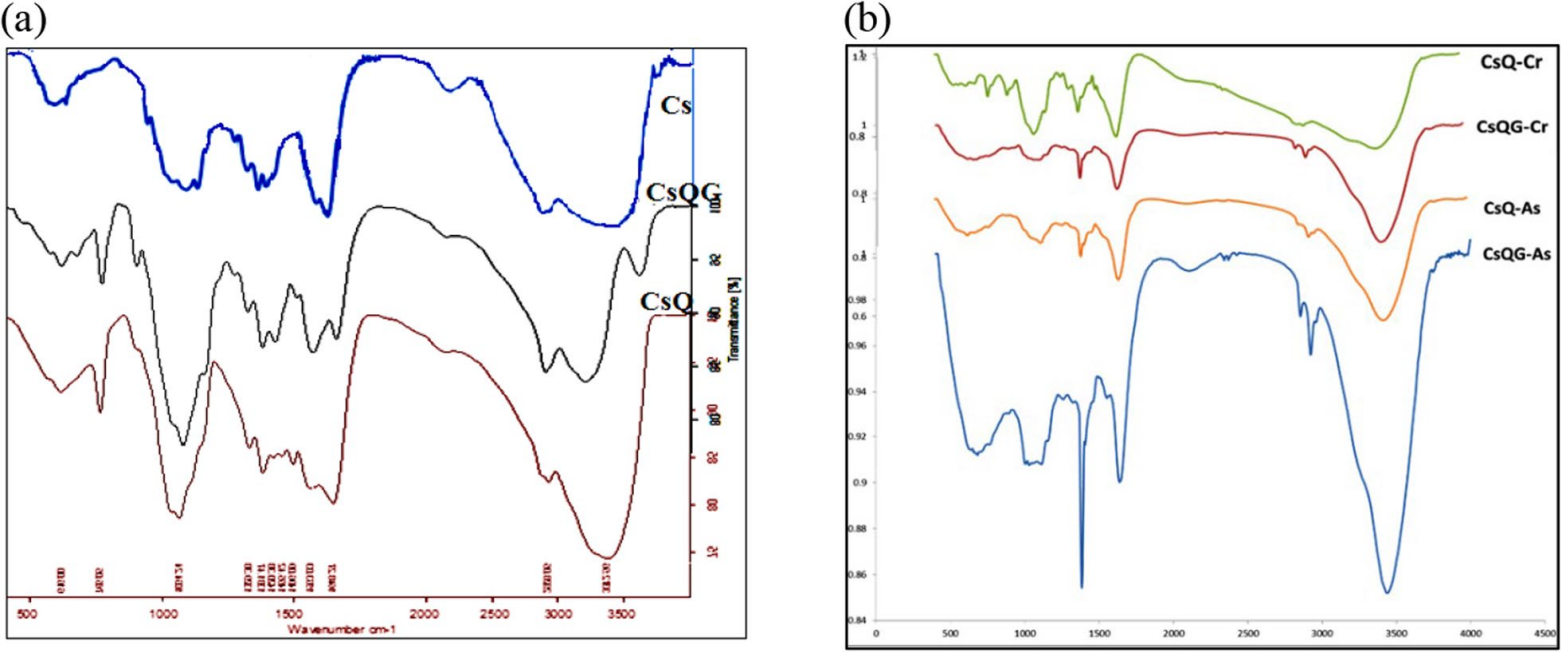
FTIR spectrum for the adsorbent before (**a**) and after (**b**) the surface absorption process

The FTIR analysis is an analytical method used to highlight the chemical structure of compounds. The FTIR spectrum of chitosan shows the characteristic peaks at 3374 cm^−1^ (O–H stretch), 2886 cm^−1^ (C–H stretch), 1660 cm^−1^ (C=O stretching), 1596 cm^−1^ (N–H bending), 1381 cm^−1^ (C–N stretch), 1157 cm^−1^ (C–O–C glycosidic link), and 1099 cm^−1^ (C–O stretch). The comparison of these spectra reveals that CsQ and CsQG spectra are quite similar to chitosan since they have the same backbone, besides the appearance of C=N at 1650 cm^−1^ and C=C aromatic ring stretching bands appeared at 1496 cm^−1^ and 1455 cm^−1^. Likely, the peaks at 896 cm^−1^, 765 cm^−1^, and 669 cm^−1^ are corresponding to = C–H out-of-plane deformation. This proves the successful modification of the parent chitosan using quinoxaline-2-carbaldehyde (via covalent bond) [45]. Also, there is no indication of the distinctive infrared band associated with the free aldehydic group near 1720 cm^−1^. On the other hand, a sequential increase in the intensity of the ethylenic bond frequency at 1564 cm^−1^ and the same behavior for the C-H stretching vibration frequency at 2886 cm^−1^ were seen in the CsQG spectrum [46]. This finding can be explained by the glutaraldehyde molecule's involvement in the CsQ glutaraldehyde reaction (Fig. 1a).

The FTIR spectral features of CsQ and CsQG following Cr(VI) and As(V) adsorption are shown in Fig. 1b, which also shows a similar type of absorption bands as that of the CsQ and CsQG. The effective adsorption of Cr(VI) and As(V) ions onto CsQ and CsQG adsorbents is concluded by the shift in the band's position observed at 1650 cm^−1^ towards the lower wavenumber (1644, 1638, 1635, and 1637 cm^−1^ in case of CsQ-Cr, CsQ-As, CsQG-Cr, and CsQG-As, respectively) corresponding to C=N stretching and the appearance of new metal-N bonds [47], nearly at 525 cm^−1^.

**Fig. 2:**
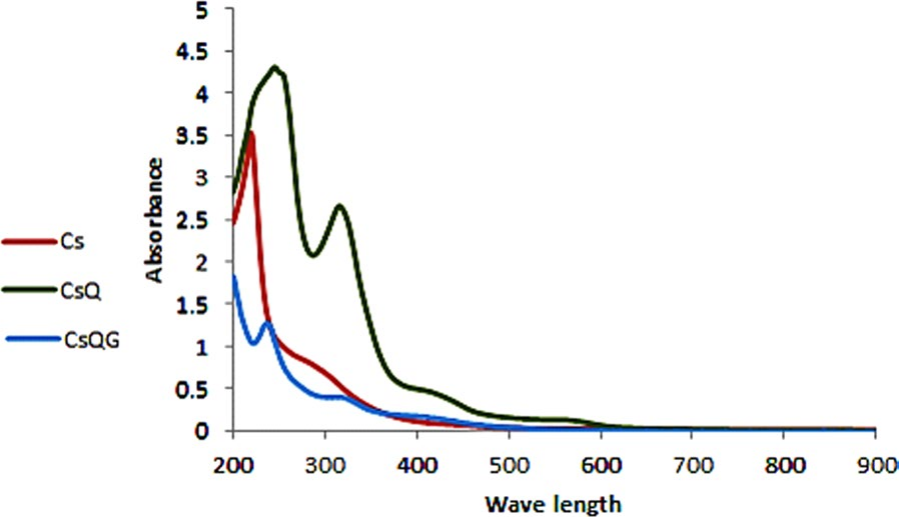
UV–Vis absorbance spectra of Cs, CsQ, and CsQG

The UV–Vis absorbance spectra of chitosan and the chitosan Schiff base (CsQ) are shown in Fig. 2. The spectrum of the parent Cs shows one peak at ƛ (200 nm) and a broad shoulder at ƛ (205 nm). In contrast, the chitosan Schiff base (CsQ) 's resulting spectrum shows two high-intensity bands assigned to π* transition at 235 and 310 nm. The latter also presents a broad shoulder band at ~ 410 nm, attributed to π* transition. This shift in the wavelength area of the modified polymer indicates the reaction occurrence, besides the variation of the shape and intensity of the peaks [48]. On the other hand, the CsQG spectrum shows three bands at 200 nm, 225 nm, and a small broad one at 305 nm, which indicates a blue shift of the CsQ after the reaction with glutaraldehyde (Fig. 2).

**Fig. 3:**
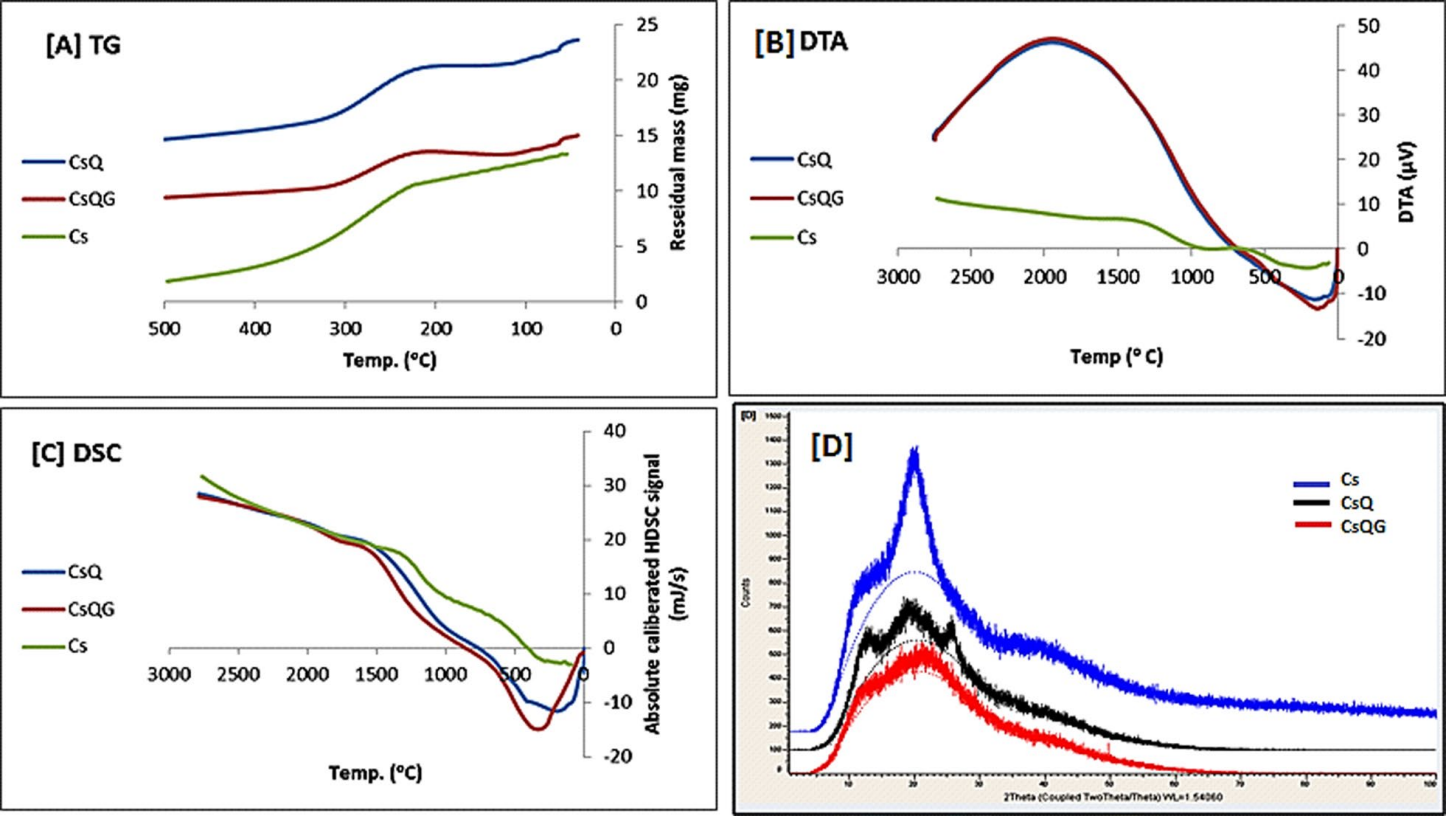
**A** TG **B** DTA **C** DSC curves of Chitosan, CsQ and CsQG** D** XRD of Chitosan, CsQ and CsQG

The XRD patterns of the CsQ show three broad peaks around 13°, 20°, and 27°. After the cross-linking, the resulting polymer CsQG showed one broad peak from 10–30°. This means that the amorphous characteristic increased. The X-ray diffraction of chitosan [49] showed two peaks: a broad one around 10° and a sharp one at θ = 20° (Fig. 3D).

To investigate the influence of the modification of chitosan on the thermal properties of the polymer, TG/DTA and DSC were used to analyze chitosan, chitosan Schiff’s base, and the cross-linked chitosan. The weights of the polymer samples were from 8 to 10 mg and were heated at 10 °C/ minute in the temperature range (30 – 500) °C.

The TG curves of Cs, CsQ, and CsQG present two stages; the 1st stage represents weight loss due to water evaporation below 100 °C. The resulting graphs reveal that the water-polymer content in Cs, CsQ, and CsQG was different, which may be due to the deference of the free amino groups' availability after the reaction with aldehyde and then with the cross-linker [50]. The second weight-loss stage was − 11.43 mg, − 7.505 mg, and − 5.827 mg for Cs, CsQ, and CsQG, respectively, which means that the weight loss amount was decreased in CsQ and CsQG compared to Cs, *i.e.,* the thermal stability of the modified polymers is better. (Fig. 3).

The DSC curve of Cs shows three endothermic peaks, while the CsQ and CsQG curves show two endothermic peaks. The midpoint of the first endothermic peak represents the value of the glass transition temperature ( Tg). The Tg of Cs, CsQ, and CsQG were 94.2, 102.9, and 78.5 °C, respectively. The point of reaction of the last stage of decomposition is higher in the modified polymers CsQ (238.5 °C) and CsQG (246.6 °C) than in Cs (204.9 °C).

**Fig. 4:**
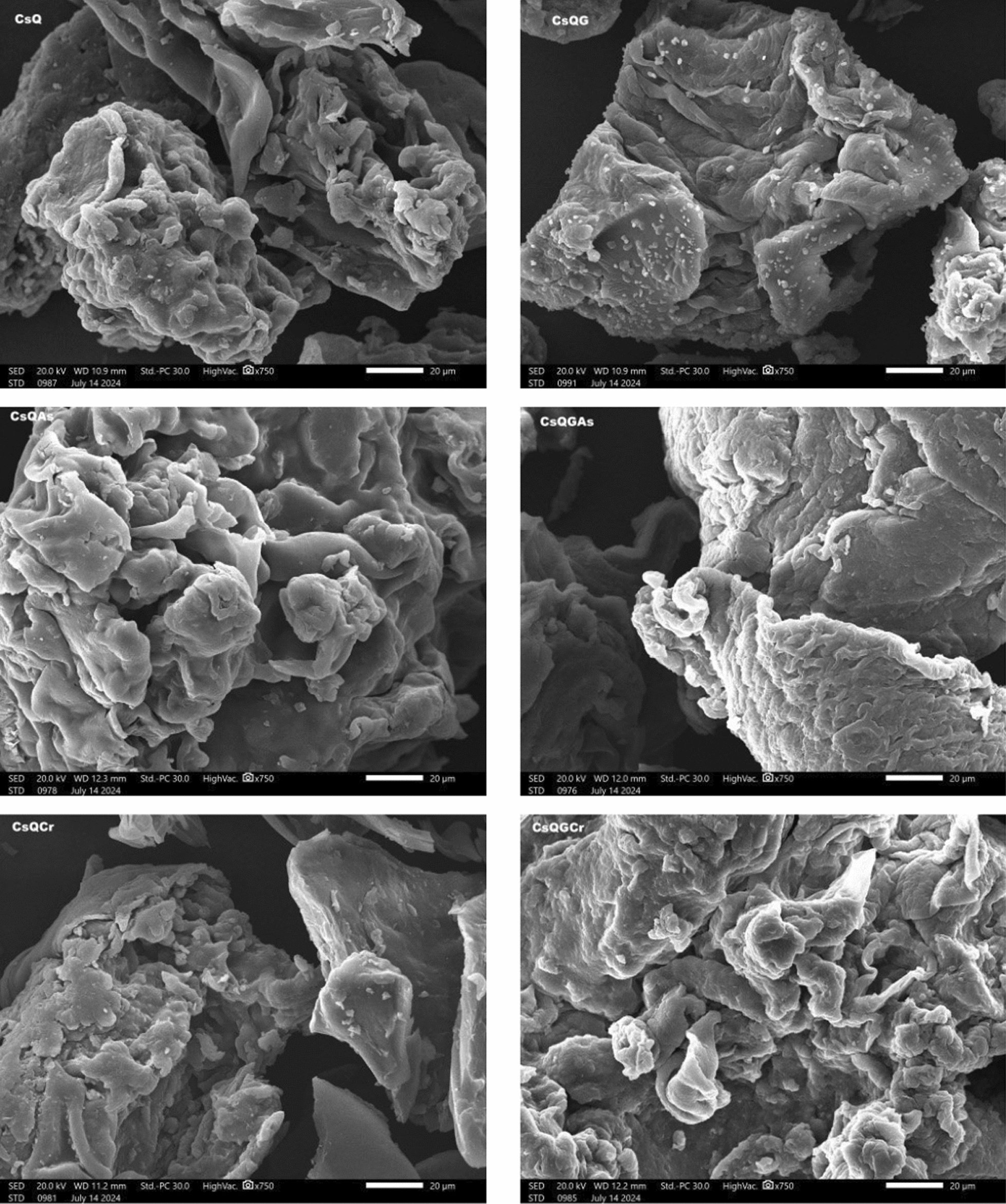
SEM images of CsQ, CsQG, CsQ-As, CsQG-As, CsQ-Cr, and CsQG-Cr

The morphology of the modified chitosan was investigated before and after crosslinking (CsQ and CsQG) using a scanning electron microscope, and the results are shown in Fig. 4. In the CsQ structure, the surface contains many wrinkles and pores, which can be suitable sites for metal ions in the adsorption process. After the modification of CsQ using glutaraldehyde as a cross-linker, significant changes were made in the surface of the CsQG polymer. The wrinkles and pores were decreased, which made the surface smoother; this could be due to the presence of glutaraldehyde (Fig. 4). The morphology of the surface after the adsorption process of As(V) ions using CsQ and using CsQG was also investigated. Some considerable changes could be a result of placing As(V) ions in the wrinkles and pores in the polymer surface (Fig. 4). In the same way, using the modified chitosan CsQ and CsQG for Cr(VI) adsorption, the SEM photo showed that there is some degree of variations.

### Adsorption studies of As(V) and Cr(VI)

#### pH influence on the adsorption process

**Fig. 5:**
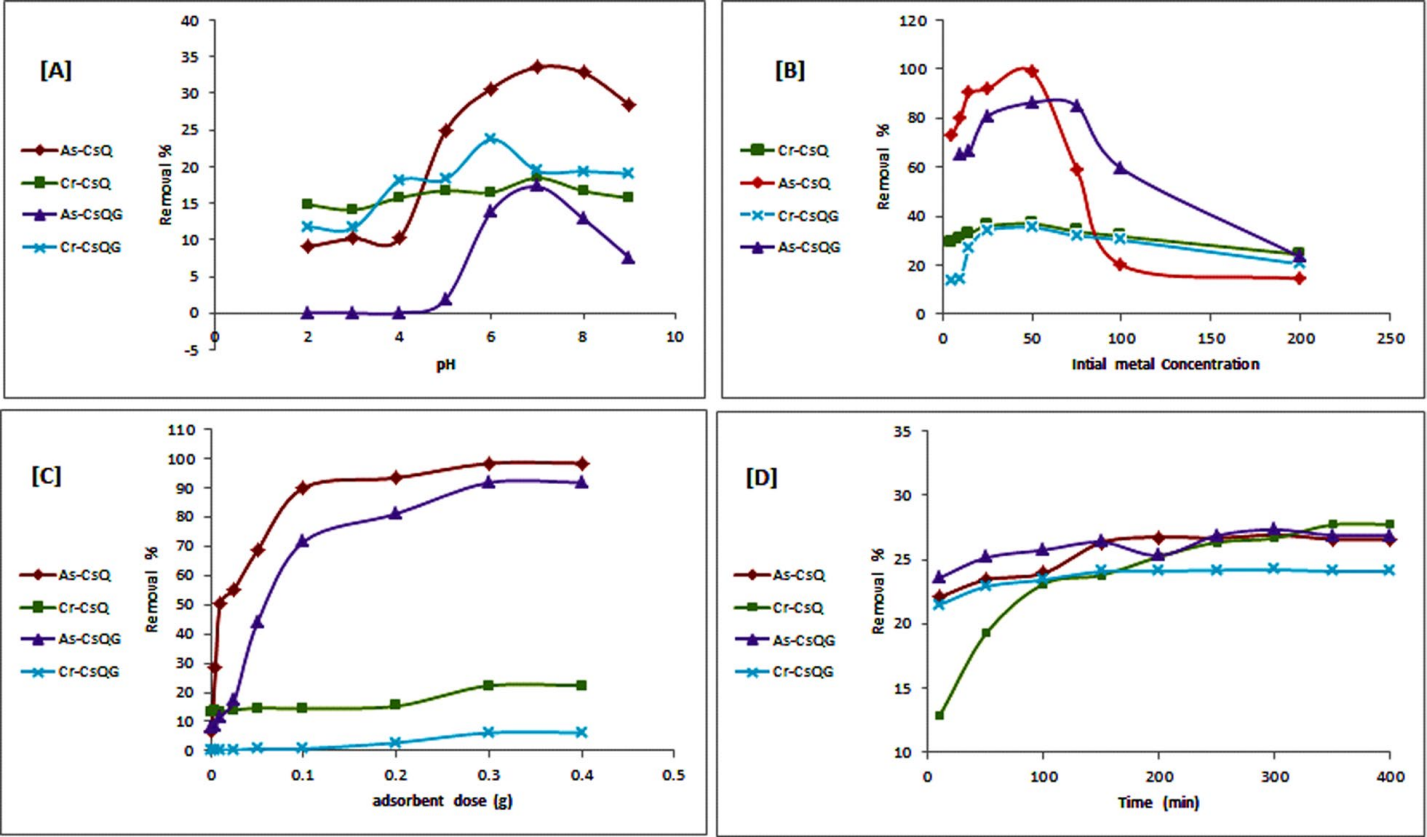
Influence of **A** pH, **B** the initial concentration, **C** polymer dose and **D** contact time on As(V) and Cr(VI) sorption

Figure 5A separately represented the pH influence on Cr(VI) and As(V) adsorption using CsQ and CsQG. Cr(VI) and As(V) adsorption were studied at a constant contact time of 60 min, using adsorbent dose (0.1 g/100 mL), initial metal concentration (200 ppm), and agitation speed (250 rpm) at room temperature. Cr(VI) and As(V) uptakes increased as the pH increased to pH = 7, and then the uptake started decreasing (*i.e.,* pH = 7 is the optimum adsorption pH). In the case of chromium adsorption using CsQG, the maximum uptake was at pH = 6, which means that the cross-linking using glutaraldehyde changed the pH of the maximum uptake to be more acidic.

The efficiency of the metal ions adsorption increased by increasing the initial pH from 3 to 6, which can be attributed to two factors; one of them is the competition between H^+^ and Cr(VI) or As(V) ions on the adsorbent active sites, and the other factor is the presence of H^+^ reduction in the aqueous solution [51]. Metal ion uptake was noted to be less at pH (2–4) because the protonated sites on the adsorbent were increased. Figure 5A indicated that the adsorption of As(V) is more than Cr(VI), which may be because As(V) has higher surface reactivity than Cr(VI). This can be attributed to the difference in the oxidation states of the elements. This difference may cause As(V) to form stronger ionic bonds with the surface of the adsorbing material. Additionally, the smaller size of the As(V) cation allows it to penetrate pores and crevices in the adsorbing material more easily [52]. This allows more surface area for As(V) adsorption than for Cr(VI). These factors all contribute to the higher adsorption of As(V) over Cr(VI).

**Fig. 6:**
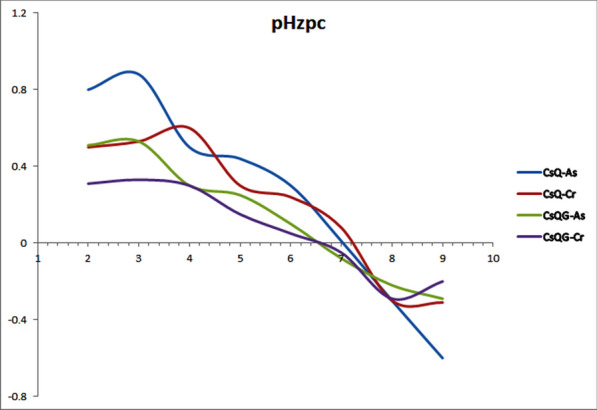
Determination of zero-point charge (pHzpc)

The pH of the solution affects the adsorbent surface charges, consequently affecting the efficiency of the Cr(VI) and As(V) adsorption process. Therefore, the surface charge of adsorbents at different pH levels should be determined and measuring the zero-point charge (pHzpc) is important. The pHzpc parameter indicates the pH where the charge of the adsorbent surface is zero. The results (Fig. 6) show that the pHzpc factor was about 7 for CsQ and around 6 for CsQG. According to that, If the modified polymer is used in a solution that has a lower pH or higher than the pHzpc values, the charge will turn positive or negative, respectively [53].

The polymer surface will be positive at pH values less than pHpzc, preventing positively charged Cr^6+^ or As^5+^ ions from approaching it. The interaction of Cr^6+^ ions or As^5+^ ions with the polymer surface may decrease because H^+^ ions compete with them, which explains the low adsorption efficiency at low pH values.

As pH rises, pH > pHpzc will happen, and the polymer surface will become negative. The negative polymer surface interacts electrostatically with Cr^6+^ or As^5+^ ions, which means that the adsorption efficiency increased^54^.

#### Initial concentration influence

The chromium and arsenate initial concentrations influence removal efficiency by considering 5, 10, 15, 25, 50, 75, 100, and 200 ppm. Figure 5B shows that at low initial concentrations (from 5 to 50 ppm) of Cr(VI)and As(V), the removal efficiency increased until reaching the maximum at 50 ppm. Following that, the metal removal decreased in both cases using CsQ [from 36.9% to 24.4% for Cr(VI) and from 98.9% to 14.5% for As(V)] and using CsQG [from 35.4 to 20.5% for Cr(VI) and from 86.4 to 23.3% for As(V)]. Such removal decline after 50 ppm may be due to the decreasing of the polymer active sites compared to the metal ions since, at low metal ion concentrations, the ratio of the metal ions in the solution to the free active sites of the adsorbent is high. Also, the metal ions can interact easily with the adsorbent (modified polymer) and can be removed from the aqueous solution since the mechanism of adsorption is that the metal molecules can form chemical bonds with the polymeric surface due to the presence of functional groups on the polymeric material (such as OH, NH_2_, C=N, C=O and aromatic ring) which increase the adsorption rate [44], as they can form chelation bonds with metal ions. This increases the likelihood of successful adsorption. Besides, we can notice that the adsorption of As(V) is better than Cr(VI), which means that the sort of metal ion can have a beneficial effect on the adsorption capacity of the cross-linking polymer.

#### Polymer dose influence

The adsorbent dose is one of the important parameters that specifies the adsorption ability and the adsorption capacity of the metal ions [6]. The effect of polymer dosage on metal ions' adsorption from water has been extensively studied, and it has been observed that the increase in polymer dosage leads to an increase in metal ions' adsorption efficiency [55]. Thus, polymer adsorbents can better enhance the adsorption of heavy metal ions in water [52]. This has important implications for treating water contaminated with metal ions, as polymer adsorbents can provide an effective solution. In the present work, the polymer dose variation was studied for both chromium and arsenate removal separately, at constant parameters (pH = 7, 100 mL of the metal solution, metal concentration of 200 ppm, and one hour contact time) and by considering various polymer dose as 0.001, 0.005, 0.01, 0.025, 0.05, 0.1, 0.2, 0.3 and 0.4 g/L. The results show that the removal rates for both Cr(VI) and As(V) increase as the polymer masses rise from 0.001 to 0.3 g/L; however, after that, the removal efficiency is nearly constant (Fig. 5C). This increasing pattern can be attributed to the availability of the free sites on the surface [56]. The CsQ polymer optimum dose for Cr(VI) removal is 0.3 g/L (22.3%), and in the case of As(V) removal is 0.3 g/L (98.4%). Whilst the CsQG optimum dose is 0.3 g/L (6.2%) and (91.7%) for Cr(VI) and As(V) respectively, which indicates that introducing glutaraldehyde into CsQ net doesn’t enhance the polymer adsorption activity (Fig. 5C).

#### Contact time influence

Contact time has a significant impact on metal adsorption on the polymer surfaces. We assess the effect of varying contact times (in the range of 10–400 min) on the Cr(VI) and As(V) adsorption. This was done using an adsorbent dose of 0.1 g/100 mL, an initial metal concentration of 200 ppm, pH = 7, and an agitation speed of 250 rpm at room temperature. The optimal removal efficiency of Cr(VI) and As(V) using CsQ is reached within 350 min and 300 min, respectively (Fig. 5D). On the other hand, the optimal removal efficiency using CsQG is reached at 300 min (24.2%) and (27.3%) for Cr(VI) and As(V), respectively. The longer the contact time, the higher the metal adsorption rate on the polymer surface. This is because the longer a metal is in contact with a surface, the more opportunities it has to form strong chemical bonds with that surface [57]. Metal-polymer interactions also become stronger, leading to higher adsorption rates.

Figure 5D shows that in both cases either using CsQ or CsQG, Cr(VI) removal is less than As(V) removal, as well, CsQ is better adsorbent than CsQG. Cross-linking with a high percentage uses a high number of amino groups, which causes a deficiency of the free (NH2) groups, subsequently decreasing the polymer reactivity for reactions. Additionally, the closed environment of the beads makes it more difficult for reactants to reach the polymer chains, which can cause the polymer chains to lose their flexibility [58].

### Adsorption isotherm studies

#### Langmuir sorption isotherm

Langmuir isotherm models were used for the experimental adsorption data fitting using the equation:

2 $$\frac{{C}_{e}}{{Q}_{e}}=\frac{1}{{K}_{L} {Q}_{m}} +\frac{{C}_{e}}{{Q}_{m}}$$

where C_e_ is the adsorbate equilibrium concentration (mg/L), Q_e_ and Q_m_ are the adsorption quantity and the maximum adsorption capacity (mg/g), and K_L_ is the adsorption constant of Langmuir. Q_e_ was calculated using the following equation:

3 $${Q}_{e}=\frac{\left({C}_{0}-C\right)V}{W}$$

where V is the volume of the solution (L), W is the adsorbent weight (g), C_0_ and C are the initial concentrations and final concentration (mg/L).

#### Freundlich Adsorption Isotherm

The heterogeneity of the adsorbent surface is represented by the Freundlich model by plotting log Q_e_ vs. log C_e_. Both K_f_ (Freundlich constant) and 1/n (Freundlich exponent) were calculated from the graph slope and intercept using the following:

4 $$\text{log}{Q}_{e} =\text{log}{K}_{f}+\frac{1}{n}\text{log}{C}_{e}$$

Q_e_ is the equilibrium adsorbed quantity (mg/g), and C_e_ is the metal equilibrium concentration (mg/L).

#### Temkin isotherm model

The Temkin isotherm model basically assumes that the heat of adsorption of all the molecules in a layer decreases linearly due to the increase in the adsorbent's surface coverage. The Temkin model also takes into consideration the effect of indirect adsorbate-adsorbent interaction on the adsorption process.

The following equation represents the Temkin isotherm model:

5 $${q}_{e}= \frac{RT}{ b}\text{ln}{K}_{t}+(\frac{RT}{b})\text{ln}{C}_{e}$$

K_T_ is the equilibrium binding constant (L/mol) corresponding to the maximum binding energy, b is related to the adsorption heat, R is the universal gas constant (8.314 J/K/mol), and T is the temperature at 298 K. The constants K_T_ and b can be calculated from the slope (RT/b) and intercept ($\frac{RT}{ b}\text{ln}{K}_{t}$) Of the plot of q_e_ versus ln C_e_ (Fig. 9).

#### Dubinin–Radushkevich isotherm model

The Dubinin–Radushkvich model has been offered as another isotherm model which is not based on the assumption of homogeneous surface or constant adsorption potential but is generally applied to express the adsorption mechanism with a heterogeneous surface and predict the adsorption process nature (physical or chemical). The following equation can represent this model:

6 $$\text{ln}{q}_{e}=\text{ln} {q}_{m}- \beta {\varepsilon }^{2}$$

where β (mol^2^/J^2^) is the Dubinin–Radushkvich constant, and ɛ is the Polanyi potential, which can be determined from the following equation:

7 $$\varepsilon =RTln (1+\frac{1}{{C}_{e}} )$$

*R* is the universal gas constant (8.314 J/mol. K), and *T* is the temperature (K). The values of D–R constants, *β* and *q*_*m*_, were calculated from the plot of ln *q*_*e*_ versus ɛ^2^ (Fig. 10).

*β* value states the average free energy (*E*), which can be calculated using the following Eq.  [51]:

8 $$E=\frac{1}{\sqrt{2\beta }}$$

**Fig. 7:**
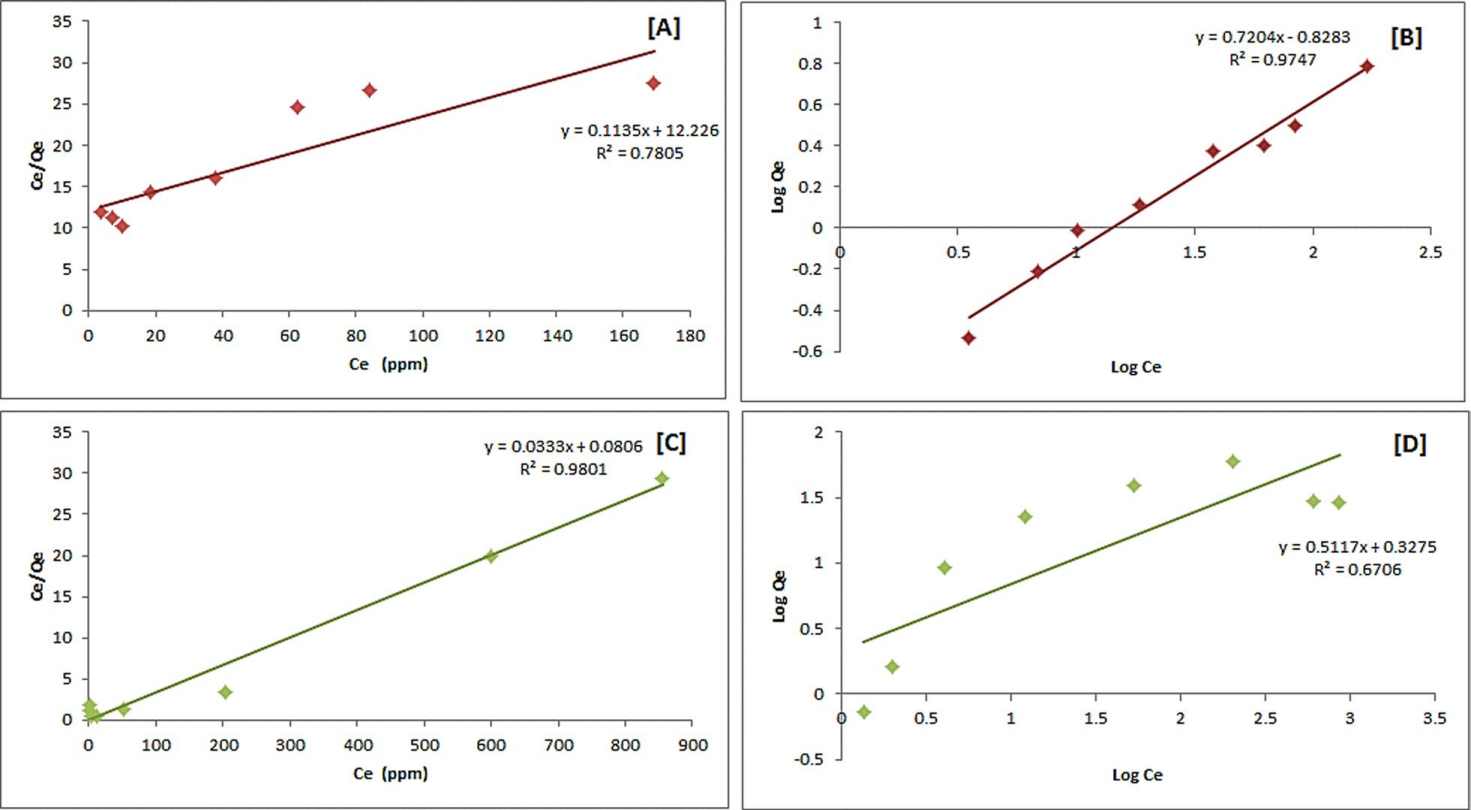
Langmuir and Freundlich isotherm linear plots: **A** and **B** for As(V) adsorption, **C** and **D** for Cr(VI) adsorption using CsQ

**Fig. 8:**
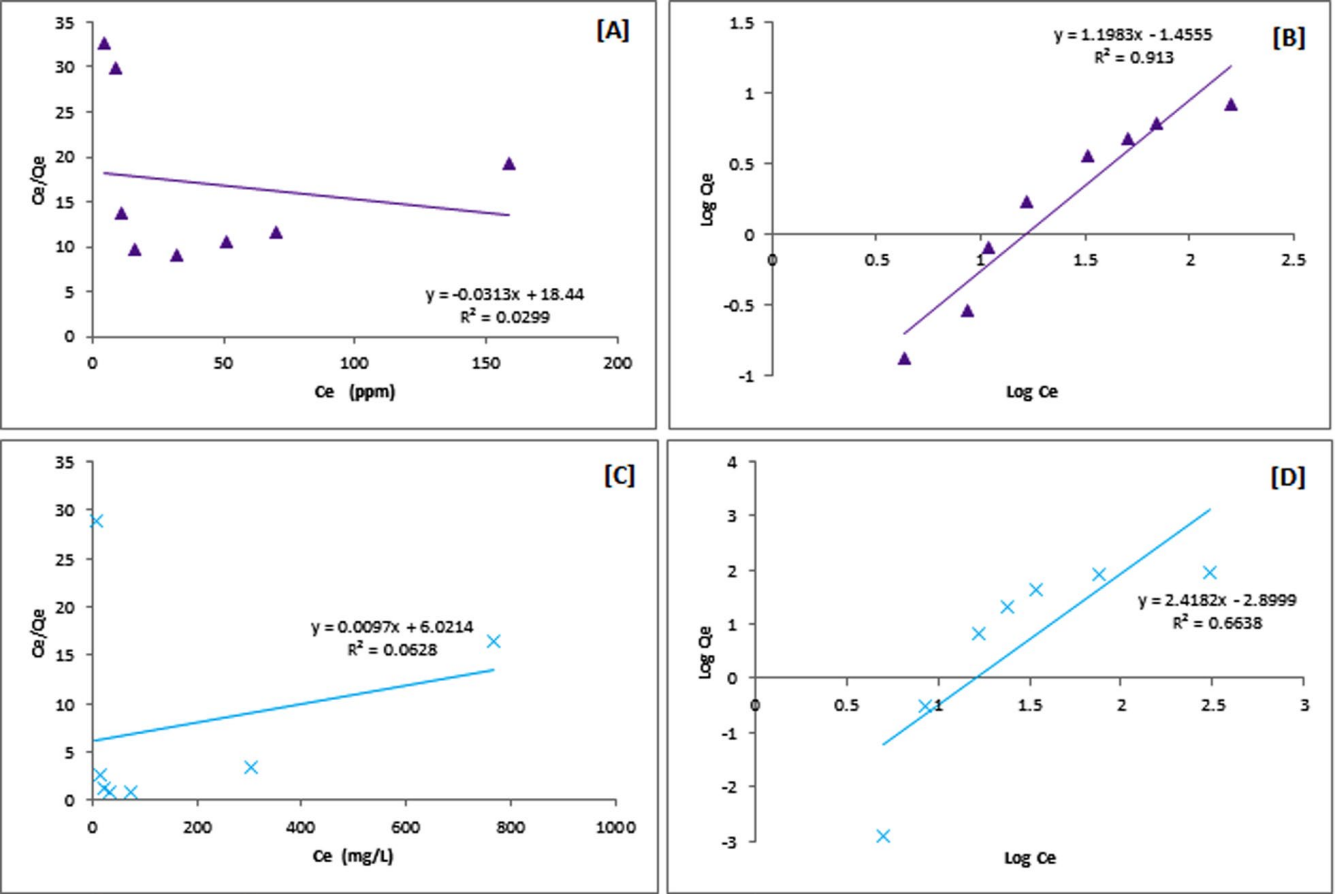
Langmuir and Freundlich isotherm linear plots: **A** and **B** for As(V) adsorption, **C** and **D** for Cr(VI) adsorption using CsQG

The Langmuir (by plotting C_e_/Q_e_ vs. C_e_) and Freundlich (by plotting log Q_e_ vs. log C_e_) adsorption isotherms are presented in Fig. 7 (using CsQ) and Fig. 8 (using CsQG). The metal concentrations (10–1000 mg/L) at pH = 7 and 1 g of adsorbent were set. In the case of using CsQ for As(V) adsorption, the Freundlich isotherm model shows a better linear fit (R^2^ = 0.9747) than Langmuir (R^2^ = 0.7805) (Fig. 7A, B). On the contrary, the Langmuir model is better fitting (R^2^ = 0.9801) than Freundlich in the case of Cr(VI) (Fig. 7C, D) (*i.e.,* monolayer adsorption took place). In addition, in the Cr(VI) and As(V) adsorption by all studied adsorbents, the R_L_ values range between 0 and 1 (Table 1), which means that the adsorption process is favorable [56].

**Table 1:** Adsorption isotherm parameters of Langmuir and Freundlich of arsenate and chromium adsorption

Polymer	Metalion	Langmuir model				Freundlich Model		
		*Q*_*m*_ (mg/g)	*K*_L_ (L/mg)	*R*^2^	*R*_*L*_	*1/n*	*K*_F_ (L/mg)	*R*^2^
CsQ	As	8.811	107.72	0.7805	0.0090 × 10^–3^	0.7204	6.7344	0.9747
CsQ	Cr	3.003	0.2420	0.9801	4.0000 × 10^–3^	0.5117	2.1257	0.6706
CsQG	As	31.95	589.16	0.0299	0.0017 × 10^–3^	1.1983	0.0350	0.9130
CsQG	Cr	103.09	620.75	0.0628	0.0016 × 10^–3^	2.4182	0.0013	0.6638

The corresponding parameters are listed in Table 1. The results show that the maximum sorption capacity Q_m_ for As(V) reached 8.811 mg/g, and for Cr(VI) reached 3.003 mg/g. In the case of using CsQG, the Freundlich model yields a better linear fit (R^2^ = 0.9130) and (R^2^ = 0.6638) compared with the Langmuir (R^2^ = 0.0299) and (R^2^ = 0.0628) for As(V) removal and Cr(VI) removal, respectively (Fig. 8). From the resulting data, the maximum sorption capacity for As(V) on CsQG reaches 31.95 mg/g, and for Cr(VI) reaches 103.09 mg/g (Table 1).

Table 1 presents the resulting *n* values from Freundlich isotherm in the range of (0.5–2.4), which indicates that the interactions between the metal ion and the adsorbent occurred. This parameter also expresses the adsorption type in detail; the *n* values higher than 1 indicate favorable and physical adsorption, as in the case of using CsQ as adsorbents for both Cr(IV) and As(V) with n values equal to 1.383 and 1.954 for As(V) and Cr(IV), respectively. While the *n* values less than 1 indicate favorable and chemical adsorption [59, 60] as in the case of using CsQG as adsorbents for both Cr(IV) and As(V), with n values equal to 0.835 and 0.414 for As(V) and Cr(IV), respectively.

**Fig. 9:**
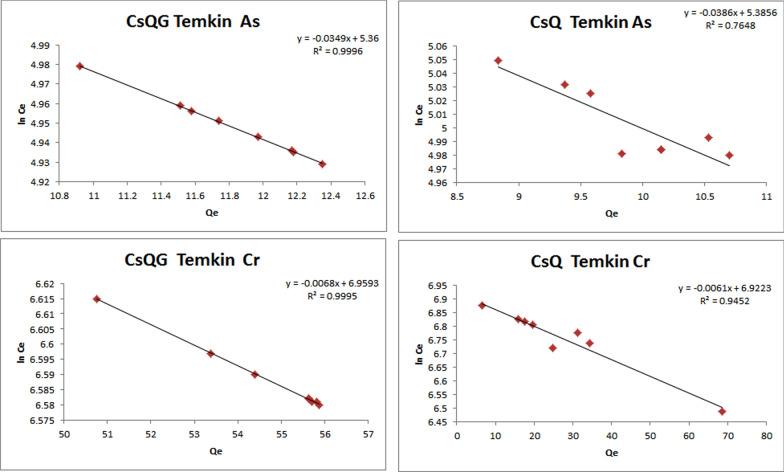
Temkin isotherm linear plots for As(V) adsorption, and for Cr(VI) adsorption using CsQ and CsQG

**Fig. 10:**
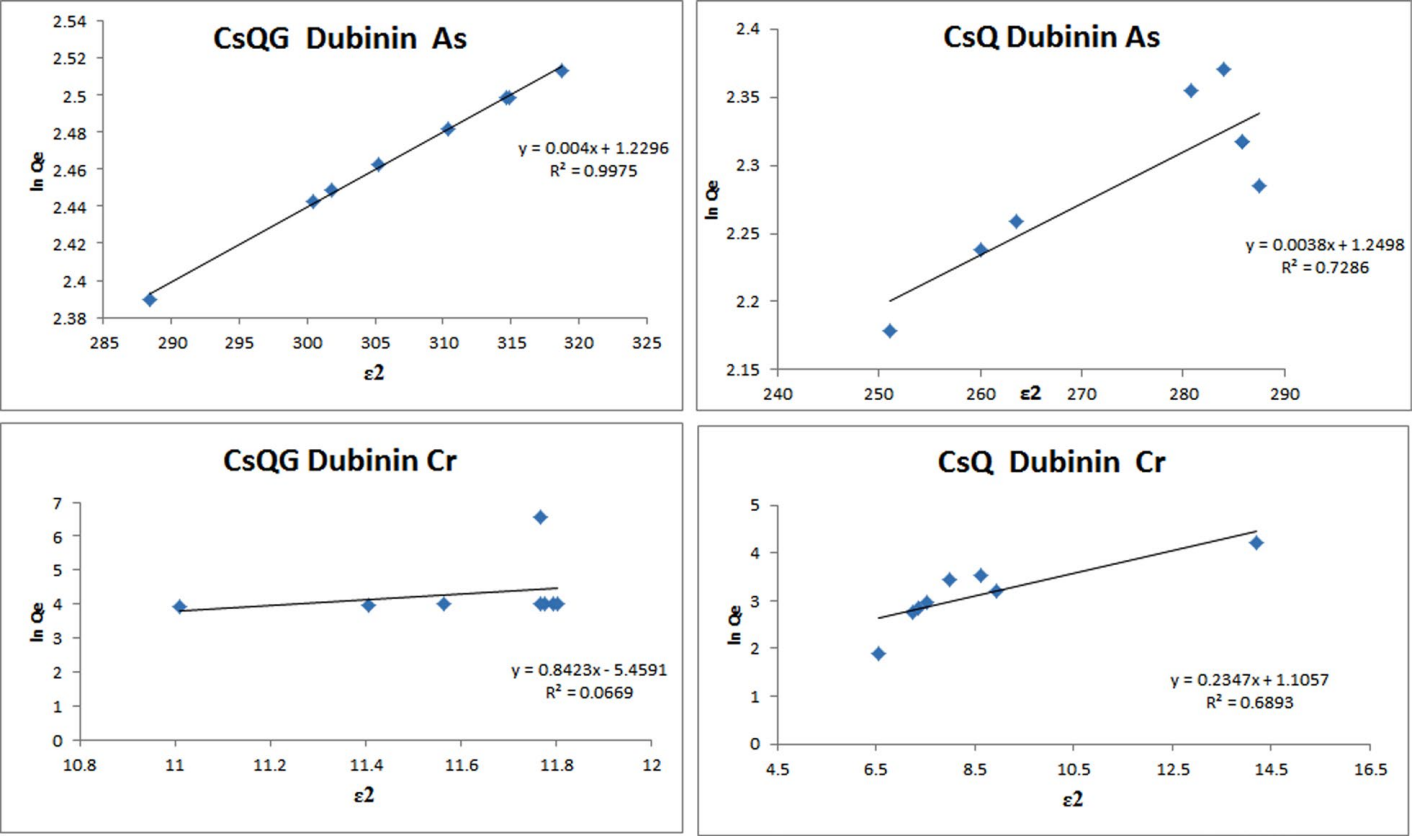
Dubinin–Radushkevich isotherm linear plots for As(V) adsorption, and for Cr(VI) adsorption using CsQ and CsQG

The linear plot graphs of both Temkin isotherm and Dubinin–Radushkevich isotherm models are presented in Fig. 9 and Fig. 10. Comparing the two resulting plots of the arsenate removal using CsQ, it was found that the higher R^2^ value results from the Temkin isotherm (0.7648) than the Dubinin–Radushkevich isotherm (0.7386); indicating better applicability of Temkin isotherm model. Likewise, the Temkin isotherm model is the more fitted for chromium ions removal with R^2^ = 0.9991. Furthermore, the Temkin isotherm model is the most applicable (R^2^ = 0.9996) for arsenate removal using CsQG, also chromium removal using CsQG, which is fitted on the Temkin isotherm model with R^2^ = 0.9991 (Table 2).

**Table 2:** Adsorption isotherm parameters of Temkin model and Dubinin model of arsenate and chromium adsorption

Polymer	Metal ion	Temkin model				Dubinin–Radushkevich model			
		*B (mol*^*2*^*/J*^*2*^*)*	*b*_*T*_* (kJ/mol)*	*K*_*T*_* (L/g)*	*R*^*2*^	*E (kJ/mol)*	*Q*_*m*_* (mg/g)*	*Β or K*_*ad*_* (mol*^*2*^*/J*^*2*^*)*	*R*^*2*^
CsQ	As	5.385	460.0	0.9928	0.7648	11.47	3.489	0.0038	0.7386
CsQ	Cr	6.922	357.9	0.9991	0.9552	1.459	3.021	0.2347	0.6893
CsQG	As	5.360	462.2	0.9935	0.9996	11.18	3.419	0.0040	0.9975
CsQG	Cr	6.959	356.5	0.9990	0.9995	1.770	0.004	0.8423	0.0669

The average adsorption free energy *E* helps in the adsorption nature indication. If the *E* value is in the range (8–16) KJ/mol, then the adsorption process is chemical, and if the *E* value is less than 8 kJ/mol, the adsorption process can be considered as a physical process [61]. The *E* values from Dubinin–Radushkevich isotherm are reported in Table 2. The *E* values equal 11.47 and 1.459 kJ/ mol for As(V) and Cr(IV) removal using CsQ. On the other hand, the *E* values are equal to 11.18 and 1.770 kJ/ mol for As(V) and Cr(IV) removal using CsQG. In summary, the *E* values indicate chemical adsorption for As(V) removal and physical adsorption for Cr(IV) removal.

Unlikely, the maximum adsorption capacity Q_m_ for As(V) and for Cr(VI) is equal to 8.811 and 3.003 mg/g, respectively, using CsQ (Table 1). Such Q_m_ values indicate ineffective removal of these ions, which wasn't expected compared to the reported literatures [62–65]. However, after cross-linking modification using glutaraldehyde, the maximum sorption capacity Q_m_ of CsQG is enhanced to 31.95 and 103.09 mg/g. (Tables 3 and 4).

**Table 3:** Comparing the adsorption capacities of the “CsQ and CsQG” adsorbents with literature data

Adsorbent	Qm (mg/g) for Cr(VI) removal
CsQ	3.003
CsQG	103.09
brown algae Sargassum bevanom [69]	40.06
Hydrous iron oxide/aluminum hydroxide composite loaded on CFA [70]	33.3
inorganic clays modified magnetic chitosan adsorbent (ICMMCA) [71]	94.67
Chitosan/β-cyclodextrin beads [72]	400
chitosan/poly(ethylene oxide)/permit electrospun nanofibers (CS/PEO/PT) [73]	208
quaternized date palm waste (QDPW) [74]	22.26
nano-MoS2/GO nanocomposite [75]	43.95
active carbons (ACs) [76]	26.25
BSC [77]	24.6
carbon nanotubes (CNTs)	
Rice husk [78]	30
mesoporous carbon microsphere (MPCMS) [79]	165.3
GO-NiFe LDH [80]	53.6
TCMR [81]	27.04
L-cysteine-doped polypyrrole-modified bentonite (L-cys/PPy/BT) [82]	318.5

**Table 4:** Comparing the adsorption capacities of the “CsQ and CsQG” adsorbents with literature data

Adsorbent	Qm (mg/g) for As(V) removal
CsQ	8.811
CsQG	31.95
chitosan beads [83]	1.90
Chitosan-iron oxide composites [84]	22.47
iron oxide Magnetite [85]	3.65
chitosan-magnetite (ChM) hydrogel beads [86]	66.9

The main benefit of using a cross-linking polymer for the removal of As(V) and Cr(VI) ions is that it gives the adsorbent higher mechanical strength [66], which increases its stability and makes it easier to handle. Although the adsorption capacity is not very promising, and many adsorbents yielded significantly higher than the said value, the presented modified chitosan has several benefits, such as its high mechanical stability, flexibility, ability to operate effectively across a wide pH range, low cost, wide availability, and long life.

Additionally, the high sorption capacity of the cross-linking polymer can be enhanced by adjusting the cross-linking concentrations of the polymer (glutaraldehyde %), which can be beneficial in removing higher amounts of ions [67]. Furthermore, the cross-linking polymer has favorable adsorption energy, which helps capture the ions more effectively. Lastly, the cross-linked polymer has a homogeneous structure, which helps in improving the rate of diffusion of ions into the polymer and provides a uniform adsorption surface [68] for the adsorbed ions.

### Kinetics studies

The adsorption process kinetic parameters were investigated by monitoring As(V) and Cr(VI) removal percentages within 10—400 min as contact time. The following equations represent pseudo-first-order and pseudo-second-order kinetics (9, 10).

9 $$\text{ln}\left({Q}_{e}-{Q}_{t}\right)=-{K}_{1}t+\text{ln}{Q}_{e}$$

10 $$\frac{t}{{Q}_{t}}=\frac{1}{{{{K}_{2}Q}_{e}}^{2 }t}+\frac{t}{{Q}_{e}} $$

Here, Qe, and Qt are the metal adsorbed amounts (mg/g) at equilibrium and at time t (min), respectively. The values of the pseudo-first-order adsorption constant k1 (min-1) can be determined from the slope resulting from the linear plotting of ln (Qe—Q_t_) vs. time. The pseudo-second-order adsorption kinetics constant k_2_ (g mg^−1^ min^−1^) can be calculated from the resulting slope of the linear plotting of t/Q_t_ vs. time.

**Fig. 11:**
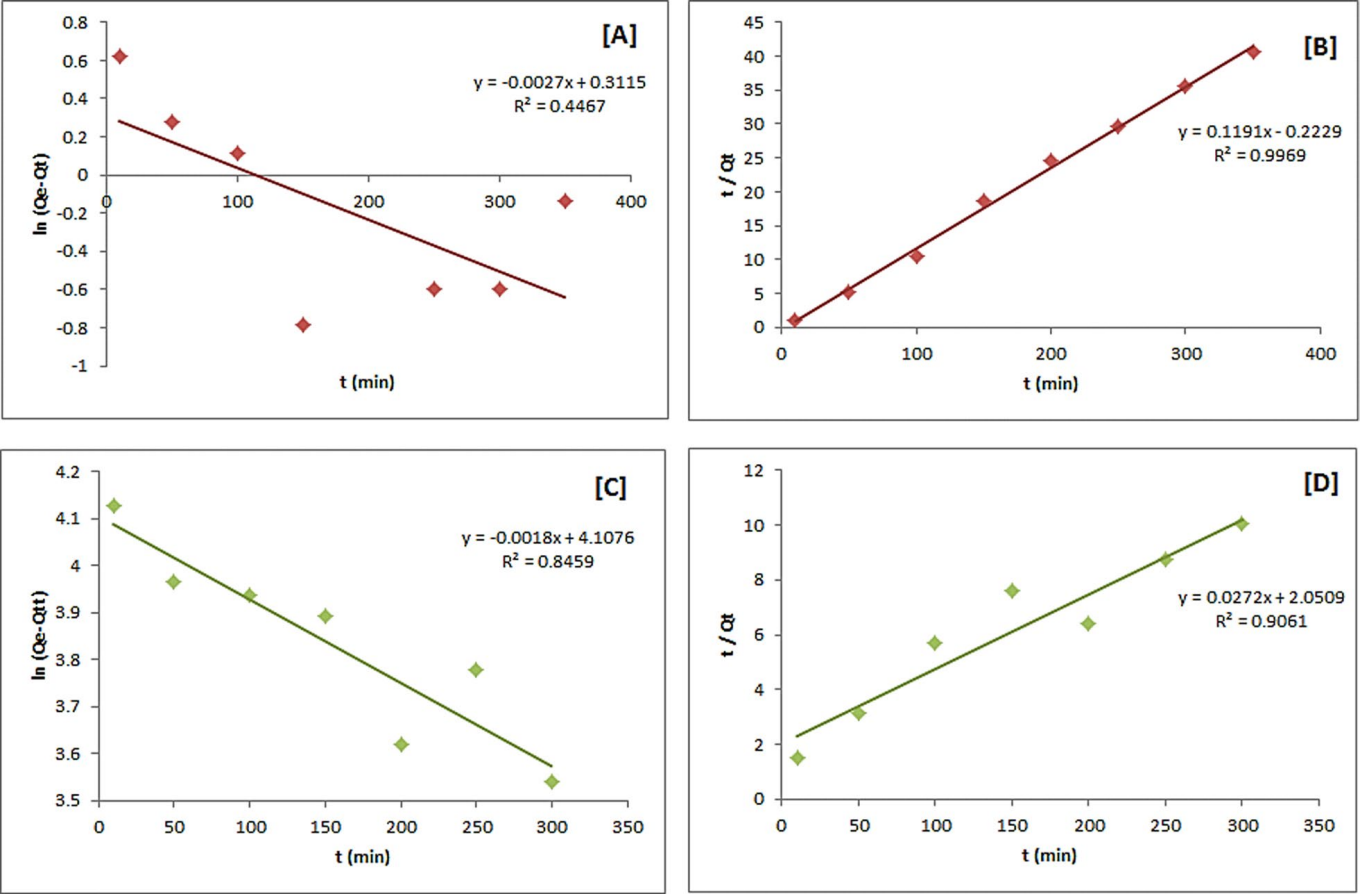
Pseudo 1st order kinetics model plots of [A] arsenate adsorption, [C] chromium adsorption and Pseudo 2nd order kinetics model plots of [B] arsenate adsorption, [D] chromium adsorption using CsQ

The linear plot graphs of both the pseudo-first-order and the pseudo-second-order models are presented in Fig. 11, as well as Table 5, summarizing the Qe, k1, k2, and R^2^ values. Comparing the two resulting plots of the As(V) removal using CsQ, it was found that the higher R^2^ value is from the pseudo-second-order plots (0.9969) than the pseudo-first-order (0.4467). This indicates better applicability of the pseudo-second-order model (*i.e.,* the adsorption mechanism is chemically rate-controlling). Likewise, the pseudo-second-order model is more fitted for Cr(VI) ions removal with R^2^ = 0.9061.

**Table 5:** Pseudo-first order and pseudo-second order constants and ***R***^**2**^ of arsenate and chromium adsorption

Polymer	Metal ion	Pseudo-first order model			Experimental value	Pseudo-second order model		
		*Q*_*e*_ (mg/g)	*k*_1_ (min^−1^)	*R*^2^	*Q*_*e*_ (mg/g)	*Q*_*e*_ (mg/g)	*k*_2_ (g mg^−1^ min^−1^)	*R*^2^
CsQ	As	1.366	0.0027	0.4464	9.830	8.396	00.064	0.9969
CsQ	Cr	60.80	0.0018	0.8459	68.66	66.77	0.0004	0.9061
CsQG	As	2.149	0.0196	0.9378	12.18	12.32	0.0195	0.9989
CsQG	Cr	42.24	0.0155	0.7433	55.87	56.18	0.0092	0.9999

**Fig. 12:**
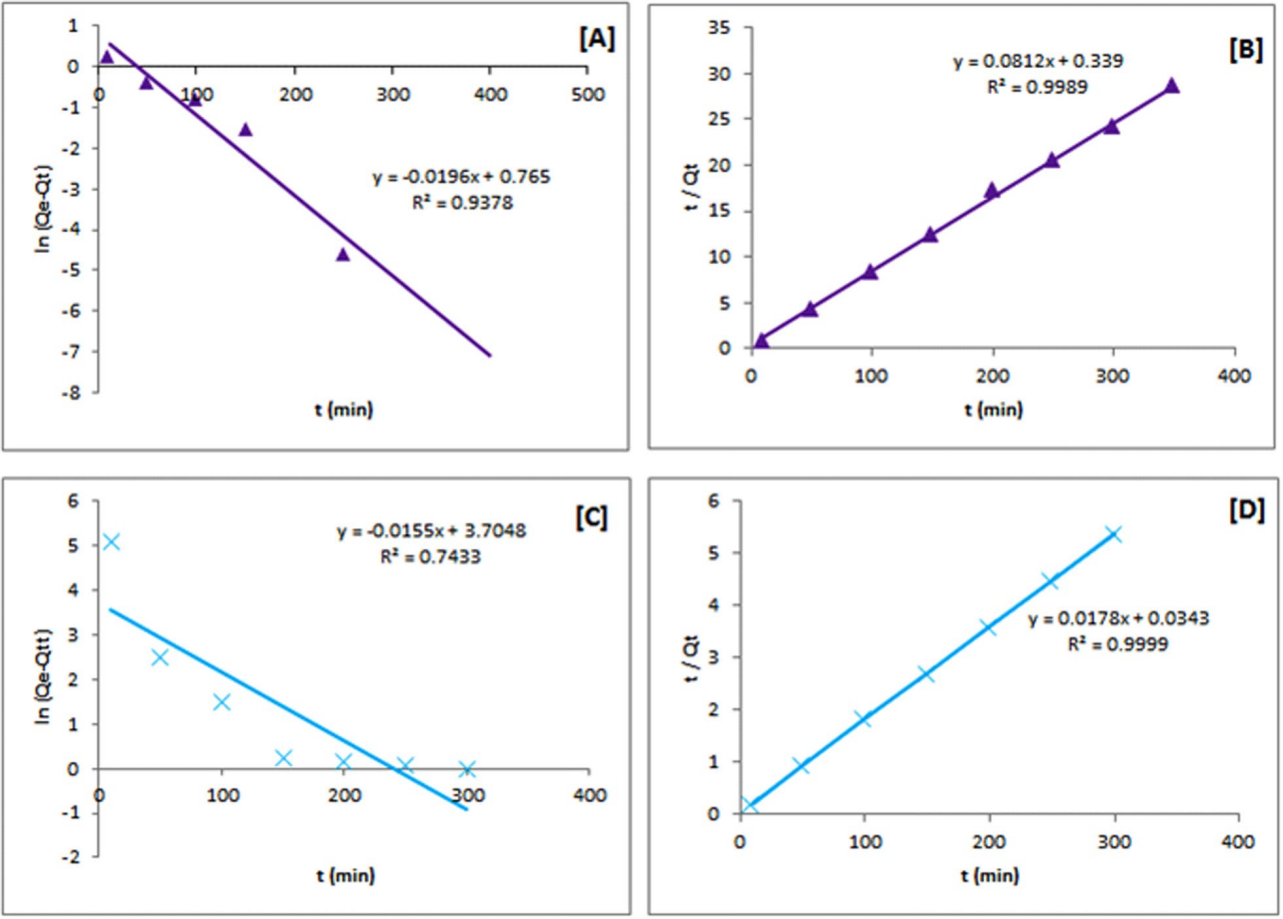
Pseudo 1st order kinetics model plots of [A] arsenate adsorption, [C] chromium adsorption and Pseudo 2nd order kinetics model plots of [B] arsenate adsorption, [D] chromium adsorption using CsQG

Figure 12A, B indicate that the pseudo-second-order model is the most applicable (R^2^ = 0.9989) for As(V) removal using CsQG. Contextually, Fig. 12C, D show the plots of Cr(VI) removal using CsQG, which is more fitted to the pseudo-second-order model with R^2^ = 0.9999.

Additionally, the resulting data showed that in the case of As(V) removal using CsQ and using CsQG, the experimental Qe values were 9.8 and 12.18 mg/g, which agree with the theoretically calculated Qt values (8.39 and 12.32 mg/g) of the pseudo-second-order PSO model. In the same way, in the case of Cr(VI) removal using CsQ and using CsQG, the experimental Qe values ( 68.6 and 55.8 mg/g) were matched with the theoretically calculated Qt values (66.7 and 56.18 mg/g) of the pseudo-second-order PSO model. The compatibility between the theoretically calculated Qt values and experimental Qe values also supports the fit of kinetics to the PSO model.

The mechanism of the removal of metal ions using modified chitosan usually includes physical surface adsorption, surface complexion, metal ion exchange, and co-precipitation. One of the effective mechanisms in adsorption is the electrostatic force created between the active groups in the adsorbent with the metal ions. There are –OH, C–O, C–H, C–O–C, N–H, NH_2_ and C–C in the polymer structure. These functional groups can interact with the metal pons in the aqueous solution and remove it through complexation and cation-π interaction mechanisms. Also, physical adsorption through pores on the polymer surface can remove the metal ions through the van der Waals forces [87].

### The reusability (regeneration) of the adsorbent

Removing the metal that has been biosorbed from the biosorbent is called recovery or desorption. In order to minimize reliance on a steady supply of biomass, maintain low process costs, and guarantee the recovery of metal ions stuck to the solid phase, biosorbent recovery is crucial. Leaching with diluted acids is a widely used practical technique for removing heavy metals from the surface of the polymer. This is so because ion exchange mechanisms are present in most metals. Metal ions separate from the polymer surface when the acidity of the metal-polymer increases.

The reusability of CsQ and CsQG in the Cr (VI) and As(V) adsorption was investigated in the present study. The resulting data showed that CsQ and CsQG have a high ability for metal ion removal up to 6 and 7 times of reuse, and the adsorption efficiency was decreased by 10–20%.

## Conclusions

It is not easy to adsorb arsenic and chromium by cross-linking polymers. It involves several procedures, including oxidation, adsorption, and desorption. Using X-ray diffraction (XRD), thermogravimetric analysis (TGA), and Fourier-transform infrared spectroscopy (FTIR), chitosan Schiff base and cross-linked chitosan Schiff base (CsQ and CsQG) were synthesized and characterized. The results of the adsorbent characterizations proved their formation where the appearance of both the C=N band and the C=C aromatic ring band in the FT-IR spectra and the disappearance of the HC=O aldehydic band confirmed the formation of the target, which was also confirmed by the variation in the XRD, UV, TGA, and SEM results comparing to the parent chitosan. The adsorption characteristics of CsQ and CsQG toward Cr(VI) and As(V) ions were studied where the Freundlich isotherm provided the best interpretation for the equilibrium data, except for using CsQ for As(V) adsorption, where the Langmuir model is better fitting. The maximum adsorption capacities for As(V) are 8.811 and 31.95 mg/g, while for Cr(VI), they are 3.003 and 103.09 mg/g on CsQ and CsQG, respectively. For all systems examined, the adsorption kinetics followed the mechanism of the pseudo-second-order model, indicating that the adsorption mechanism is chemically rate-controlling. As(V) removal was less than Cr(VI) removal in both cases, whether using CsQ or CsQG, we finally found that CsQ is a better adsorbent than CsQG.

## Data Availability

The research data within the manuscript.
